# Robot-Assisted Surgery in Gynecology

**DOI:** 10.7759/cureus.29190

**Published:** 2022-09-15

**Authors:** Gayatri R Bankar, Ajay Keoliya

**Affiliations:** 1 Medicine, Jawaharlal Nehru Medical College, Datta Meghe Institute of Medical Sciences, Wardha, IND; 2 Forensic Medicine, Jawaharlal Nehru Medical College, Datta Meghe Institute of Medical Sciences, Wardha, IND

**Keywords:** surgeries, hysterectomy, gynecology, laparoscopy, robotic surgery

## Abstract

The advancement of robotics-based procedures in the medical industry is the subject of this review article. The purpose of the surgical robot is to increase surgical abilities and address human shortcomings. The robot's success has been predicated on its ability to accurately and consistently repeat tasks. The following are a few objectives and quantifiable benefits of robotic technology improving surgical maneuverability and physical capabilities. In 2005, the FDA granted gynecological surgery approval for the Da Vinci surgical system. It has been quickly embraced and has already taken on a significant role at many of the locations where it is offered. It consists of a cart with robotic branches next to the patient and a high-resolution three-dimensional (3D) vision system. This study covers laparoscopy via robots in benign gynecological surgeries, myomectomy surgery, hysterectomies, endometriosis, tubal anastomosis, and sacrocolpopexy. The appropriate published studies were evaluated after a PubMed search was conducted. Additionally, procedures that may be used in the future are highlighted. In benign gynecological illness, most currently available research does not show a substantial benefit over traditional laparoscopic surgery. Robotics, however, does provide help in more complicated operations. Compared to laparoscopy, robotic assistance has a lower conversion rate to open procedures. Endo wrist movement of an automated machine during myomectomy surgery can perform proper and better suturing than traditional laparoscopy. The automated program is a noticeable improvement over laparoscopic surgery and, if price issues are resolved, can gain popularity among gynecological surgeons around the globe.

## Introduction and background

Gynecologic surgeons have expanded marginally interfering surgery into their surgical range over the last 30 years. One of the more incredible benefits of robot-assisted surgery (RAS) is the advancements such as joint-wristed tools, tremor control, and three-dimensional (3D) stereoscopic vision for sophisticated tissue viewing and manipulation. The US Food and Drug Administration (FDA) approved the widely used platform, the da Vinci Surgical System, for a limited range of gynecologic operations in 2005. The system is now the only robotics stage on the market with FDA approval. Less postoperative discomfort, enhanced surgeon ergonomics, quicker analysis of the instrumentation's curve, elimination of the fulcrum effects, and other advantages of this particular platform over traditional laparoscopy are some of its advantages and a more ordered integration of fluorescence technology for lymphovascular estimation [1].

With its high-resolution 3D perspective, wrist-like mobility of the robotic arms, and better ergonomics, it offers the benefits of superb visualization [2]. Beyond the first and second years, there is a lot of room for advancement in the surgical performance of robotic surgery. Even after many years, as seen by the decreased surgical timeframes, the surgical performance keeps getting better [3].To assess if robotic surgery's higher direct and indirect costs are justified by any advantages in patient clinical outcomes, it should be directly compared to traditional laparoscopy [4]. Important questions about the learning process are highlighted as computer-assisted laparoscopic technology usage and acceptance gain popularity. A training course for robotic surgery might encompass a variety of modalities [5]. The majority of program directors anticipated that the use of robotic surgery will grow and become more pervasive in gynecologic surgery. In facilities that offered residency training but had insufficient resources for efficient resident training, robotic surgery was extensively reported. A planned curriculum may be beneficial for robotic surgical training in obstetrics and gynecology residency, according to additional evaluation [6].

This speedily developing technology is a substitute for open procedures and complicated laparoscopy. The assumption of robotic laparoscopic surgery into gynecologic practice in a few years has been noted. The FDA authorizes the system for gynecological conditions depending on the primary verification of welfare and potency from its initial incident with myomectomy and hysterectomies [7]. Gynecologists can use the robotic system to conduct hysterectomies, myomectomies, and lymph node bishop. In 1999, the Cleveland Clinic published the first research on robotic surgery in gynecology [8]. Robot-assisted surgery has facilitated growth in marginally interfering surgery for gynecologic problems. Although there isn't enough high-quality data to confirm, robot-assisted gynecologic surgery may take longer and have a higher risk of drawbacks.

Gynecologic oncology and the application of the da Vinci surgical system in benign gynecology are included in this category. Robotic surgery can nurse fibroids, irregular menstrual cycles, endometriosis, ovarian tumors, uterine prolapse, and various cancers in females. In the United States, robot-assisted hysterectomy appears to be much more costly than a standard laparoscopic hysterectomy for benign conditions in 2015, with no contrast in comprehensive complication rates. The Hominis robotic system (Memic Innovative Surgery Ltd., Tel Aviv, IL) seeks to develop a motorized policy for vaginal myomectomy using natural orifice transluminal endoscopic surgery [9]. According to a study of surgical excision of the uterus and cervix in the initial stages of cervical cancer, both robotic, as well as a laparoscopic surgery, had equal cancer consequences [10]. With RAS, a relatively recent advancement in laparoscopic surgery, the physician may do the procedure from a computer console that is not on the operating table. Robot-assisted surgery is a viable option for various gynecological surgeries and is already routinely utilized in the United States for hysterectomy. However, independent assessment is needed to determine whether RAS is more clinically successful and safe than traditional laparoscopic surgery (CLS) [11]. 

For a female who goes through a hysterectomy, myomectomy, sacrocolpopexy surgeries, and cancer staging of gynecology surgeries, haphazardly controlled trials and selective retroactive learning revealed the association of minimally invasive surgery with less blood loss, little clinical stay, fewer difficulties before and after an operation, and good quality of life compared with open incision surgeries [1]. Sacrocolpopexies, benign hysterectomy, myomectomy, radical hysterectomy, and lymph node dissection are examples of gynecological robotic-assisted laparoscopic surgeries. Robot-assisted gynecologic surgery sometimes takes longer in the operating room than open or laparoscopic surgery but typically has comparable clinical outcomes, less blood loss, and a shorter hospital stay [7].

## Review

Hysterectomy

Hysterectomy is one of the most typical gynecological surgeries worldwide, and an attempt has been made over the years to make it easier and less complicated with the advent of marginally interfering techniques. Still, the incidence of open hysterectomy has been raised in the USA as well as in India. The focus should be to do as many hysterectomies that are marginally interfering by vaginal or laparoscopic aspects. This gives better functional outcomes regarding decreased postoperative illness and quicker healing. The American Association of Gynecologic Laparoscopists advocated that many hysterectomies for benign conditions be carried out additionally through the vagina or by using laparoscopy.

Despite marginally interfering procedures being used for hysterectomy, RAS is not broadly carried out or received in gynecological surgery. This is because of the essential steep learning curve and perhaps its technical complexity. The presentation of a robotic platform has high rates of robotic hysterectomy with low rates of abdominal hysterectomy. It is not surprising that in the last few years, the assumption of mechanical techniques to carry out gynecology surgeries, mainly uterus removal, has quicker action than that seen with the approval of laparoscopic procedures. Although, RAS for benign gynecological illness showed that it is not related to better performance but raised the price of the procedure. Most clinical results such as loss of blood, difficulties, and clinical rest, were comparable to the robot-assisted and laparoscopic hysterectomy [1]. Vaginal hysterectomy (VH) appears to be preferable to laparoscopic hysterectomy (LH) and abdominal hysterectomy (AH) among women having a hysterectomy for benign illness because it is linked to a quicker return to regular activities. Vaginal hysterectomy should be used instead of AH if it is technically possible due to more rapid postoperative healing and fewer febrile event. Laparoscopic hysterectomy provides several benefits over AH in situations when VH is not feasible (including more fast recovery and more occasional hot events and injury and abdominal wall infections). Still, these are offset by a longer operating time [12]. The most popular forms of therapy for endometrial and cervical malignancies in their early stages are total and radical hysterectomies. Open surgery, laparoscopy, and, nowadays, marginally interfering robot-assisted surgery are examples of surgical methods.

We looked through numerous electronic databases for observational studies with a control group and randomized controlled trials published between 2009 and 2014. Our result of interest involves perioperative as well as morbidity results [13]. It is necessary to continue the research on the differences between robotic and traditional laparoscopic hysterectomy and pelvic lymphadenectomy for gynecologic cancer. In all, 98 consecutive cases of gynecologic cancer patients underwent robotic-assisted hysterectomy and pelvic lymphadenectomy during the same period, while another 98 consecutive subjects underwent conventional laparoscopic hysterectomy and pelvic lymphadenectomy were included [14]. In the outpatient context, robotic hysterectomy is practical and safe for treating endometrial and cervical cancer since >39% of patients were successfully released within 12 hours without expanding readmission. A risk classification model might be created using the several risk variables for prolonged hospitalization to increase the effectiveness of outpatient robotic hysterectomy [15].

Endometriosis

Surgery for endometriosis is tricky using laparoscopy. The adherence, the dysfunction of adnexal structures, and the low results in reproduction put more pressure on the surgeon to do an absolute and thorough job. The indication faced by females with endometriosis, such as prolonged pain in the pelvis, severe cramps with heavy blood flow, subfertility, excessive bleeding during menstruation, and abdominal bloating, are usually disabled and needs care during surgery to replace anatomy and function while all endometriotic implants are extracted. Although, after laparoscopic surgery for endometriosis, usually, space is seen as an achievement of the surgeon during the surgery. It is feasible to overcome the space by robotic assistance that gives the surgeon a complete and enlarged surgical view. Endometriosis is nowadays treated mostly by surgery via mechanical aids that depict the appropriate steps included in endometriosis clearance by using automated assisted technology. In contrast to RAS with traditional surgery for endometriosis, excluding a longer operative time, there was no specific difference in blood loss, clinical rest, and difficulties. No transformation to laparoscopy was seen. Notably, no modifications are required, and the robotic perspective may be more productive in complex surgeries with specific organ compromises. A known role of RAS in deeply penetrated endometriosis includes segmental bowel operations and extraction of nodules from the rectovaginal septum with or without rectal shaving and partial bladder operation. There is no report on specific perioperative difficulties, but the anastomotic discharge was seen. This is one of the most extensive series of robotic procedures for deep infiltrating endometriosis (DIE). A mixed method for colorectal endometriosis has involved some infertile females with serious minor intestine diseases using the da Vinci system and laparoscopic surgery. All patients have manifested disease-free operating margins. Obstruction of the ureter secondary to endometriosis was adjusted with automated laparoscopic limited ureterectomy and ureteroneocystostomy. Successful administration of endometriosis of the small intestine, bladder, and ureter in a few patients by using robotic laparoscopic surgery was also reported [1].

A prevalent condition affecting females of reproductive age is endometriosis. One of the most pervasive signs of endometriosis is pain. Surgical intervention is necessary when medicinal treatment has not succeeded, or there is a known DIE. The gold standard for identifying and treating endometriosis is laparoscopy. Robotic-assisted surgery for endometriosis is demonstrated to be possible; however, it hasn't been established that it is any better than traditional laparoscopy. Fluorescence and narrow band imaging are two other advanced imaging methods that have been researched. However, long-term clinical advantages have yet to be demonstrated [16]. Robotic technology may be used in endometriosis surgery in particular circumstances. These circumstances include heterogeneity of lesions, which makes it difficult to identify them, difficulties in precisely forecasting surgical complexity, and extended operating times for severe patients. The aim is to describe robotic surgery's current and future aspects as it pertains to endometriosis [17]. Endometrial tissue outside the uterus is a frequent benign chronic condition referred to as endometriosis. The most severe manifestation of endometriosis is DIE presentation [18]. A major development that has opened up new possibilities for treating endometriosis, particularly DIE, is the development of robot-assisted laparoscopy (RAL). It offers several technological advantages in the management of this challenging condition, such as 3D vision, tremor filtering, and enhanced surgical ergonomics, which enhance surgical performances without extending operating times, blood loss, intra or postoperative complications, and also lower the rate of conversion to laparotomy [19]. The usefulness of robots in endometriosis surgery is still in question due to the absence of randomized control studies contrasting robotic surgery to conventional laparoscopy, the current gold standard for diagnostic and therapy. The advantages of robotic surgery for endometriosis in its early stages are unclear. On the other hand, several case reports and retrospective studies have revealed that robots may be useful in the treatment of deep infiltrating and advanced stage endometriosis.

Myomectomy

To allow women to keep their uterus, the surgical method of abdominal myomectomy was first defined in 1931. An identical procedure was performed laparoscopically in 1979. The success of endoscopic surgery served as the foundation for the development of robotic myomectomy (RM), which was accepted by surgeons. Since automated platforms offer many benefits, they are particularly advantageous to surgeons with few or no laparoscopic expertise, particularly in suturing procedures. Myomectomy is a suture-intensive procedure, and robotic arm aid performs suturing quicker and in a straightforward manner. This is why robotic myomectomy is so popular and widely accepted. A robotic myomectomy ensures the treatment will be as successful as a traditional open myomectomy. Robotic surgery is just as accepted and safe as laparoscopic surgery [1]. The effectiveness of robots in endometriosis surgery is still up for debate because there aren't any randomized control studies contrasting robotic surgery to conventional laparoscopy, the current standard for diagnosis and treatment. Regarding the advantages of robotic surgery for endometriosis in its early stages, there remains uncertainty. The use of minimally invasive surgery is now crucial for the surgical treatment of uterine fibromatosis [20].

Gynecology has experienced fast development and incorporation of robotic surgery. As the robot's usage in gynecological surgery was approved and found to have several benefits over traditional laparoscopy, it has been broadly used, particularly in reproductive surgery. The most prevalent benign tumors of the female reproductive system are uterine fibroids. Many reproductive-aged females had this state demand uterine-sparing surgery to preserve their fertility [21]. For treating submucosal fibroids, hysteroscopic myomectomy is a repeatable and quickly learned method. Robot assistance resolves most of the technical difficulties associated with laparoscopic myomectomy for intramural and subserosal fibroids. The combined adoption of these technologies will allow more patients with fibroids to benefit from a marginally interfering approach [22]. In comparison to the other groups, the RM group had a much lower body mass index and significantly greater uterine size, myoma diameter, and myoma weight. The majority of myomas, however, were in the open myomectomy (OM) group. Additionally, compared to the OM and laparoscopic myomectomy (LM) group, the RM group had considerably lower maximal visual analog scale values but increased operating duration and blood loss [23]. For several ablative gynecologic operations, robot aid in single-incision laparoscopy has recently been reported, and it appears to offer technical benefits. There haven't yet been any reports of this approach being used extensively with sutures. We describe an early experience with a single-incision laparoscopic myomectomy aided by a robot and offer crucial technical information to effectively reproduce this procedure in a sophisticated robotic surgical team [24].

Sacrocolpopexy

The primary cause of morbidity in women is pelvic organ prolapse, and more and more women are choosing surgical treatment over enduring discomfort and indignity. There have been 0.2% to 45% of vaginal vault prolapse cases documented. The surgeon's experience is a significant factor in determining the best course of action. The procedure made available to the patient is also influenced by the patient's fitness for surgery, age at presentation, prolapse grade, and expectations from an operation. The procedure with the highest long-term success rates is abdominal sacrocolpopexy with mesh. Robotic-assisted laparoscopy allows patients with pelvic organ prolapse who are otherwise healthy to recover quickly. Data from level III indicate that the first results of robotic sacrocolpopexy are comparable to those of open sacrocolpopexy. Robotics, laparotomy, and laparoscopy all have the advantages of precision dissection, ease of use, and safety. Robotic help, however, has the advantages of the loss of blood and shorter clinical rest. Increased vision and talent are the key benefits of the mechanical method, especially when the pre-sacral area is dissected, the mesh is placed, and intracorporeal suturing is done. During laparoscopic surgery, the use of a robot aids in the adequate performance of challenging tasks and produces positive results. Robotic sacrocolpopexy offers the benefit of making intracorporeal suturing more straightforward. The entire surgery may be assisted mechanically or just the suturing. They were contrasting with the operative time in this process variation. The first description of robotic sacrocolpopexy was published in 2004 [25]. In this study, automatic suturing aid was used, and four women had issues such as recurrent prolapse or vaginal mesh extrusion-the mean operating time in a series. In terms of age, body mass index (BMI), and concurrent surgery, patients were comparable. Statistically speaking, the automated group has statistically boosted operating times while reducing surgical blood loss and length of stay. In terms of the majority of factors, both groups' assessments of postoperative prolapse in patients were equivalent. However, as the surgeon and the team do more procedures, the operating time gets shorter. A retrospective examination of a small number of patients who underwent robotic sacrocolpopexy revealed a 25% reduction in recovery time and then a plateau [26]. The usage and advantages of automated laparoscopic sacrocolpopexy in treating post-hysterectomy vaginal vault prolapses in fatty individuals and mid-urethral sling application are described and shown in observational research from the Mayo Clinic [27]. Although robotic operations are more expensive and take longer to complete, they tend to enhance pelvic support and sexual performance [1]. The latest advancement in marginally interfering surgery is robotic surgery. It offers improved visualization and dexterity, enabling the surgeon to carry out intricate procedures beyond the scope of their capabilities with traditional laparoscopy. It would be related to high morbidity if performed by laparotomy [28].

Despite an increase in the use of robotic technology for minimally invasive hysterectomies with sacrocolpopexy, the advantages of these pricy treatments are still not well established. A study compared the expenses, 30-day readmission rates, and perioperative complications between robotic and traditional laparoscopic hysterectomy with concurrent sacrocolpopexy using a worldwide database [29]. Disorders of the pelvic floor pose a serious threat to public health. The best treatment for women's genital prolapses is sacrocolpopexy. Rectal prolapse can be treated with the relatively new and effective laparoscopic ventral mesh rectopexy procedure. There is no literature combining the two robotically assisted techniques [30]. A robotic platform is a device that has made it possible for many gynecologic surgeons to carry out treatments that would normally require a laparotomy using a less invasive method. Because of the lack of training in conventional laparoscopic suturing, knot tying, and retroperitoneal dissection, a larger proportion of hysterectomies and sacrocolpopexies were performed before the widespread adoption of this robot-assisted technique [31]. 

Abdominal sacrocolpopexy and minimally invasive sacrocolpopexy seem to be interchangeable terms. Laparoscopic and robot-assisted sacrocolpopexies appear to be just as successful as abdominal sacrocolpopexies; nevertheless, future prospective studies contrasting the long-term effects of abdominal sacrocolpopexy (ASC), laparoscopic sacrocolpopexy (LSC), and robotic sacrocolpopexy (RSC) with medical expenses are urgently needed. Advanced pelvic organ prolapse can be surgseveralected using a number of tested methods. Selection of the right surgical approach is a complex decision process involving many factors [32]. Pelvic organ prolapse (POP) is a common condition that drastically reduces women's quality of life. Practical support for the vaginal apex is a vital component of an efficient surgical repair for women with severe prolapse, including anterior and posterior wall prolapse. There are two surgical options: abdominal and vaginal. The former can be performed openly, laparoscopically, or robotically. A typical procedure for examining prolapses, including various pelvic organ prolapses and uterine or vaginal vault prolapses, is called sacrocolpopexy [33].

Other uses of robot-assisted surgery

Ophthalmology

Robotic surgery is quiet in its beginnings in the area of ophthalmology. However, there are a few robotic technologies that can successfully do surgery. The Preceyes Surgical System (Preceyes BV, Eindvitreoretinal operations that are vitreoretinal. The robot is controlled by teleoperation by ophthalmologists. This gadget mounts to the control room table, headed by a surgeon who can efficiently work due to the easy motion control. Preceyes is one of the robotic devices that has been certified by the European Commission. Foresight (Israel), Acusurgical (France), and Horizon (US) are among the other companies in this field. The da Vinci Surgical System uses telemanipulation to do pterygium procedures, despite not being designed specifically for ocular treatments such as in vivo corneal surgery [34].

Thoracic

Robotic surgery for mediastinal, pulmonary, and complicated esophageal problems has become more prevalent in thoracic surgery [35]. The da Vinci Xi system works for lung and mediastinal lump excision. Video-assisted thoracoscopic surgery (VATS) and traditional thoracic surgery, done conventionally, are two less invasive procedures that can be contrasted. Despite VATS being the shorter exorbitant choice, the robotic-assisted perspective provides benefits such as three-dimensional vision with higher expertise and coable perioperative end results [36].

Gastrointestinal

In comparison to laparoscopic pancreatectomies, robot-assisted pancreatectomies were shown to have a range of procedures, including bariatric surgery and cancer gastrectomy [37]. Initially, case papers were published by surgeons from several universities, illustrating various methods and the practicality of gastrointestinal surgery by using robotic equipment [38]. Particular therapies have been widely researched, such as esophageal fundoplication for the investigation of GERD and Heller myotomy for the investigation of achalasia [39]. A prolonged operating time, under projected loss of blood, an increased rate of spleen preservation, and few clinic stays. In terms of transfusion, transformation to open procedure, the overall difficulty, serious problems, pancreatic fistula, serious pancreatic fistula, ICU rest, gross fee, and 30-day mortality, there was no determined difference between the two categories [40].

Heart

The following are various samples of cardiac enucleation helped by robotic surgical systems: atrial septal defect, repair of the mitral valve, and bypass of the coronary arteries [41].

Spine

Spine robotic devices were first used in marginally interfering spine surgery in the 2000s. In 2014, there was a preliminary randomized clinical inquiry to assess if robot-assisted surgery of the spine is safer than other choices [42]. As of 2019, robots are used mainly in spine surgery to insert pedicle screws for spinal fixation. Furthermore, research on robotic spine surgery has focused on the lumbar or lumbosacral vertebrae. The use of robots to insert screws in the cervical and thoracic vertebrae has received little research [43].

Bone

Bone robots are utilized in orthopedic surgery. The Robodoc (Curexo Technology, Fremont, CA, USA) is the earliest dynamic robotic system to perform various surgical duties during complete hip replacement surgery (THA). It has information from computer tomography (CT) scans pre-programmed into it. After that, the surgeon may decide on the ideal size and outline for the new hip. The THA uses Acrobat and Rio, two semi-active robotic devices. The surgeon supervises the drill bit, but the mechanical system prohibits movement outside the predetermined parameters [44]. Mazor X (Medtronic Pvt Ltd., Dublin, IE) is a gadget that assists surgeons in implanting pedicle screws in spine surgeries. The wrong placement of a pedicle screw might out turn into neurovascular injury or build defeat. Using templating imaging, Mazor X locates the target spot where the pedicle screw is necessary [45].

Surgical Transplantation

The introductory robotic kidney transplants were carried out in the late 2000s. Obese people who would usually be unable to have a kidney transplant may now be able to do so. Losing weight, on the other hand, is an excellent beginning step [46].

General Surgery

Currently, robotic surgery is the most acceptable match for single-quadrant operations, which may be brought about on any of the four quadrants of the abdomen. Although strategies like cholecystectomy and fundoplication are costly, they allow doctors to hone their robotic surgical expertise [47].

Urology

Computer-aided surgery has become more common in urology, notably in the United States. There is inadequate verification of welfare in contrast to conventional surgery to warrant the more fabulous prices [48]. Few researchers have confirmed early that prostatectomy surgery results in complete cancer elimination and fewer side effects. In 2000, the earliest robotic-accommodated laparoscopic radical prostatectomy was carried out. Robot-assisted surgery has besides been used to do radical cystectomies. A 2013 review establishes fewer issues and superior short-term results compared to the open method [49].

Colorectal surgery

Robotic-assisted surgery has numerous benefits over laparoscopic colorectal surgery, including multi-arms that can be controlled by the surgeon and devices that can spin and bend in all directions. In rectal or sigmoid colon cancer treatment, high dissection and the selective ligation approach appear to reduce the anastomosis leakage rate and produce favorable clinical and oncologic results. Under robotic aid, it is simple to preserve the left colic artery and ligate the sigmoid or superior rectal artery [50].

Thyroid surgery

The established remote-access thyroid surgery, robot-assisted transaxillary thyroidectomy, is just as safe and efficient as its time-tested traditional clamp-and-tie cousin. However, it has been blamed for several problems, which surgeons must be aware of and carefully address [51].

Future scope

Robotic surgery can be fully automated by using AI and advanced imaging if the robot itself can plan and perform a surgery based on the patient's age group, gender, and risk factors such as hypertension, diabetes and other complications. Suppose the robot can plan surgery with incision size, depth of incision, types of sutures, materials, and the additional necessary navigation provided by the surgeon. Surgical robots have aided secluded surgery, marginally interfering surgery, and unmanned surgery. The surgery is carried out with accuracy, miniaturization, and tiny cut, resulting in less blood loss, pain, and speedy healing. Far from ordinary manipulation and three-dimensional magnification, articulation allows for improved ergonomics. Shorter clinic stays, less loss of blood, more periodic blood exchange, and less pain medication use are all benefits of these treatments [52]. Restricted access to the operational site, lengthy healing times, lengthy operating hours, loss of blood, surgical discoloration, and spots are all disadvantages of the current open surgery approach [53]. Compared to the prior marginally invasive surgery perspective, robotic surgical surgery gives the surgeon more command over the surgical equipment and more pleasing sight of the operating area. Furthermore, surgeons are not required to be at length and are less likely to feel exhausted. Natural hand trembles are blocked by using the robotic computer operating system. Eventually, the surgical robot may operate endlessly by rotating surgery teams [54]. Robotically operated laparoscopic camera setup occurs far more stable than human assistance, with fewer unintentional movements [55].

Table 1 shows the various advantages and disadvantages of RAS. Advantages such as three-dimensional visualization, no tremors during surgery and telesurgery, minimal scarring, reduced risk of infection, and less blood loss and transfusion as well as some disadvantages such as the expense and need for special education and training, regular approval, big size of the robotic system and lack of versatility.

**Table 1:** Advantages and disadvantages of robot-assisted surgery

Advantages	Disadvantages
Three-dimensional visualization	Expensive
No fulcrum surgery	Absence of touch sensation
No tremors during surgery	Extra time interval for surgery set up
Seven-degree of freedom	Unpredicted malfunction
Tele-surgery	Special education and training
Reduced risk of infection to the patient	Cost of maintenance
Less blood loss and transfusion	Need for regular approval
Minimal scarring	Big size of the robotic system
Less trauma to patients' body	Not versatile

## Conclusions

Robotic surgery is practiced in many places across the globe. This way of advanced assisted technology ensures no shaking of hands during the surgical procedure, minimum blood loss, and scar after surgery due to fewer open wounds. Hospital stay for the patient is reduced due to the fast-healing process and quick recovery. It is safe for the patient. Robotic surgery ensures the surgery is done with precision and accuracy. Robot-assisted surgery is one of the good options for women who go through myomectomy, hysterectomy, and pelvic organ prolapse surgery. Robotic surgery is not only included in gynecology but also other fields such as neurosurgery, orthopedic surgery, colon endoscopy, benign prostate surgery, urology, general surgery, respiratory surgery, and cardiac surgery. Robotic surgery upgrades and corrects several growing conditions and helps reduce the risk of infection. It is a fast-growing technology that is a good alternative to open surgical procedures.
